# RCSB Protein Data Bank tools for 3D structure-guided cancer research: human papillomavirus (HPV) case study

**DOI:** 10.1038/s41388-020-01461-2

**Published:** 2020-09-16

**Authors:** David S. Goodsell, Stephen K. Burley

**Affiliations:** 1 Research Collaboratory for Structural Bioinformatics Protein Data Bank and Institute for Quantitative Biomedicine, Rutgers, The State University of New Jersey, Piscataway, NJ 08854 USA; 2 Department of Integrative Structural and Computational Biology, The Scripps Research Institute, La Jolla, CA 92037 USA; 3 Department of Chemistry and Chemical Biology, Rutgers, The State University of New Jersey, Piscataway, NJ 08854 USA; 4 Research Collaboratory for Structural Bioinformatics Protein Data Bank, San Diego Supercomputer Center, and the Skaggs School of Pharmacy and Pharmaceutical Sciences, University of California, San Diego, La Jolla, CA 92093 USA; 5 Rutgers Cancer Institute of New Jersey, Rutgers, The State University of New Jersey, New Brunswick, NJ 08903 USA

**Keywords:** Target identification, Virology

## Abstract

Atomic-level three-dimensional (3D) structure data for biological macromolecules often prove critical to dissecting and understanding the precise mechanisms of action of cancer-related proteins and their diverse roles in oncogenic transformation, proliferation, and metastasis. They are also used extensively to identify potentially druggable targets and facilitate discovery and development of both small-molecule and biologic drugs that are today benefiting individuals diagnosed with cancer around the world. 3D structures of biomolecules (including proteins, DNA, RNA, and their complexes with one another, drugs, and other small molecules) are freely distributed by the open-access Protein Data Bank (PDB). This global data repository is used by millions of scientists and educators working in the areas of drug discovery, vaccine design, and biomedical and biotechnology research. The US Research Collaboratory for Structural Bioinformatics Protein Data Bank (RCSB PDB) provides an integrated portal to the PDB archive that streamlines access for millions of worldwide PDB data consumers worldwide. Herein, we review online resources made available free of charge by the RCSB PDB to basic and applied researchers, healthcare providers, educators and their students, patients and their families, and the curious public. We exemplify the value of understanding cancer-related proteins in 3D with a case study focused on human papillomavirus.

## Introduction

Function follows form in biology. Knowing the structure of a protein (or nucleic acid) is crucial for understanding how it works in nature, how it may affect plant, animal, or human health, and how it could be targeted (or harnessed) to improve the human condition. The Protein Data Bank (PDB) is the global archive of three-dimensional (3D) biomolecular structure data [1, 2]. Founded in 1971 with just seven protein structures as the first open-access digital data resource in all of biology, the PDB today houses more than 168,000 structures of proteins, DNA, RNA, macromolecular machines, viruses, and virus-like particles. The US-funded Research Collaboratory for Structural Bioinformatics Protein Data Bank (RCSB PDB; RCSB.org), a founding member of the Worldwide Protein Data Bank (wwPDB) partnership [3], integrates the entire corpus of PDB data with ~40 external biodata resources, and provides easy-to-use web-based search and molecular visualization tools to assist many millions of PDB data consumers worldwide in finding, analyzing, and visualizing 3D structures of macromolecules and their complexes with one another, drugs, antibodies, enzyme cofactors, etc.

This wealth of structural information is particularly useful in the field of cancer biology, wherein changes in DNA sequence that manifest themselves at the level of protein 3D structure and biochemical function can have extreme consequences for human health and disease [4]. Structural information is also central in the search for new approaches to diagnostics and therapeutic interventions, as we seek to block or modify these changes and their oncogenic effects [5, 6]. The PDB archive currently holds structural information covering all aspects of cancer biology, ranging from the molecular details of oncogenic mutations, to mechanisms of important cellular processes such as apoptosis, to structural characterization of the molecular machines underlying organism-scale processes such as neovascularization and metastasis. For the avoidance of doubt, current PDB holdings include 3D structures of the protein targets for >90% of the 79 new antineoplastic agents [54 small molecules, 25 biologics] approved by US Food and Drug Administration 2010–2018 [6]. In this review article, we use human papillomavirus (HPV) as a case study to highlight several diverse examples from this continuously growing corpus of 3D biostructure information, and the powerful tools available from the RCSB PDB for making effective use of them in the field of cancer research.

## Fundamental biology: revealing and understanding the HPV proteome

HPV are nonenveloped double-strand DNA viruses that infect epithelial cells. Infections with most HPV types lead to self-limiting benign lesions (a.k.a. warts), but several sexually transmitted genital high-risk types (e.g., HPV16 and HPV18) cause cervical carcinomas—the leading cause of death among female cancer patients worldwide [7, 8] —and some head and neck cancers [9]. The HPV genome encodes two classes of proteins: six early nonstructural regulatory proteins (denoted with “E” names), and two late structural proteins L1 and L2 [10]. Macromolecular crystallography (MX), NMR spectroscopy (NMR), and, increasingly, cryo-electron microscopy (3DEM) have all been used to determine 3D structures of viral proteins of HPV and related papillomaviruses, and their interactions with host proteins (Table 1). Some of these structures provide critical insights into the architecture of the papillomavirus capsid, composed of the L1 and L2 proteins, and explain how HPV virus-like particles can elicit an immune response and be recognized by antibodies that neutralize the virus. Other 3D structures reveal the atomic details pertaining to the function of the HPV early proteins, including those of E1 and E2 and their roles in viral replication, and those of E6 and E7 as they recognize and bind to intracellular proteins and frustrate host tumor suppressors, leading to oncogenic transformation.

**Table 1:** Structures of papillomavirus biomolecules in the PDB.

Experimental method		
63	Macromolecular crystallography (MX)	
14	Solution NMR (NMR)	
14	Electron microscopy (3DEM)	
Structures of papillomavirus proteins		
28	L1	13 icosahedral capsids and 10 complexed with monoclonal antibody
8	E1	3 complexed with DNA
25	E2	4 complexed with DNA
22	E6	17 complexed with host protein
8	E7	3 complexed with host protein
Structures related to oncogenic subtypes		
13	HPV16	
11	HPV18	

The results from advanced search (June 08 2020) of “papillomavirus” in “Source Organism Taxonomy Name,” with Boolean AND of additional “Full Text” terms such as “L1.”

As the PDB archive is growing at the rate of about 10% per year, it has become increasingly challenging to navigate and utilize the available holdings. To ensure that the PDB archive is maximally utilized, the wwPDB and the RCSB PDB are committed to the findability, accessibility, interoperability, and reusability principles [11] emblematic of responsible data resource management. Of critical importance, PDB structural information is available open access from members of the wwPDB partnership with no limitations on usage. Building on this freely available data repository, the RCSB PDB provides a wide range of search, analysis, and molecular visualization tools to provide nimble, multimodal access to >166,000 structures. All 3D structures coming into the PDB are processed by the wwPDB global deposition, validation, and biocuration system known as OneDep [12]. Every one of these structures is validated against both experimental data and established stereochemistry to provide users with quantitative estimates of structure quality and accuracy [13, 14]. In addition, every one of these structures is annotated by a professional wwPDB biocurator to a common data standard that was established by the wwPDB in consultation with community stakeholders [15]. Strict compliance to the PDBx/mmCIF data standard [16], rigorous structure validation, and expert biocuration ensures that our data consumers who are not experts in structural biology can rely on the information they download. The PDB has been recognized as a Core Certified Repository by CoreTrustSeal (coretrustseal.org). This international, community-based, nongovernmental, nonprofit organization promotes sustainable and trustworthy data infrastructures of which the PDB is widely regarded as a gold-standard exemplar.

**Fig. 1:**
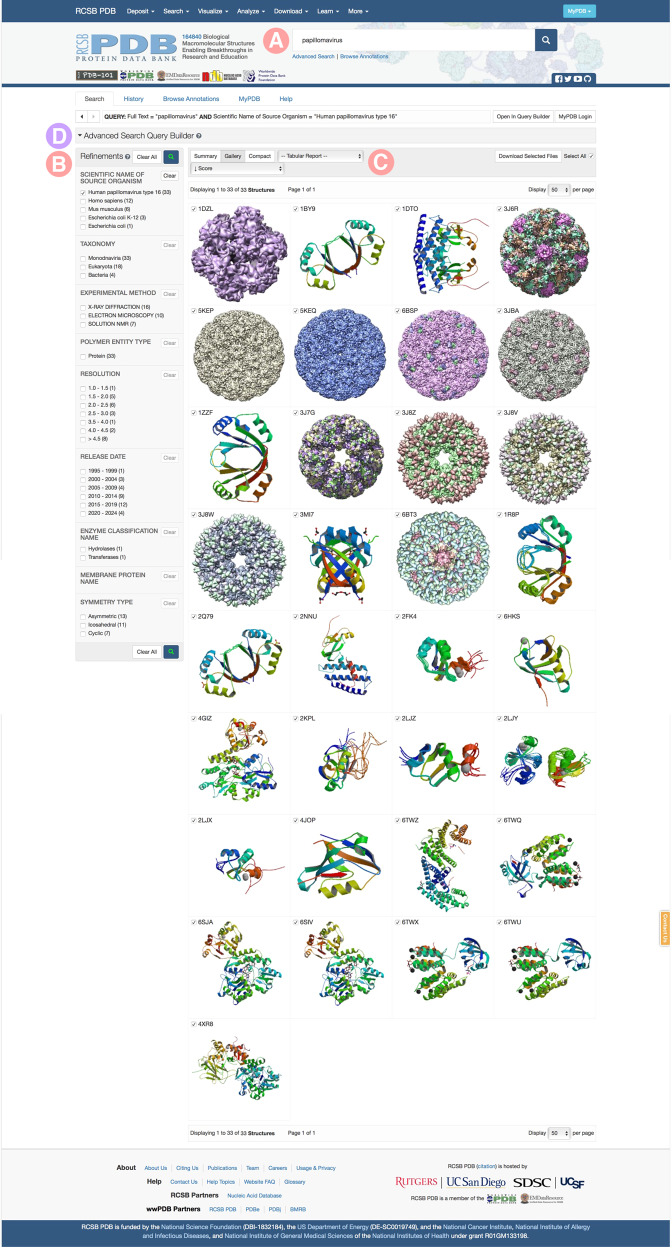
Multiple options streamline searches of the PDB archive. Here, HPV16 holdings were found by (**a**) searching for “papillomavirus” in the main search box, **b** selecting HPV16 in the refinement options, and **c** choosing a gallery display. Advanced searches may be built-in (**d**) for more specific queries. The search returns several L1 capsid structures, some coated with antibody Fab fragments, and structures of E2 and E6, some interacting with host cell proteins.

Search tools are optimized to help PDB data consumers (hereafter users) find molecules that are relevant to a given research question. These tools are hierarchical, allowing users to apply increasing levels of specificity as needed. Most begin with the general search box that is prominently displayed at the top of the RCSB PDB website home page (rcsb.org), which combines the open source Apache Solr platform with indexing of all PDB data. It provides a listing of hits scored and ordered by relevance to the user’s search term. For example, a search of “papillomavirus” in the “Source Organism Taxonomy Name” category yields 91 entries, including HPV proteins and nucleic acids, molecules from related papillomaviruses, and host proteins associated with HPV proteins. Users may then turn to Refinements to narrow this group of search hits. In Fig. 1, filtering by “Human papillomavirus type 16” narrowed the list to structures related to this particular oncogenic subtype. A flexible Advanced Search Query Builder allows interactive construction of Boolean Operator combinations of searches on a variety of subject fields, including polymer sequence, sequence motif, structure similarity, and chemical structure. This finer-grained searching for topics was used to obtain listings of each of the viral proteins summarized in Table 1. Finally, the resultant lists may be examined using a variety of textual and graphical reports, which are linked to detailed *Structure Summary* pages for each PDB structure.

**Fig. 2:**
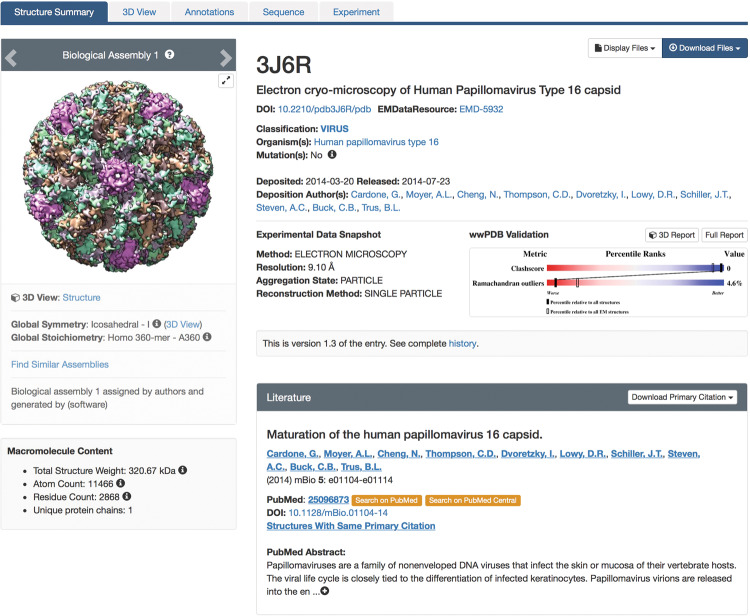
Structure Summary pages provide an overview for each entry in the PDB archive. *Structure Summary* for a cryo-electron microscopy study of HPV16 capsid (PDB ID 3j6r [19]) provides an overview of the entry and many options to access detailed information and analysis tools.

Given the state of structural biology and the enormity of the PDB archive, typical searches will return dozens of structures related to a given topic. Each PDB structure has a dedicated *Structure Summary* page that provides a telegraphic overview, which is particularly useful when filtering a selection of structures for use in a given application. These pages deliver the major features of each PDB structure, identified with a unique four-character code (e.g., PDB ID 1abc), including a static image created with the RCSB PDB Mol* web-native molecular graphics tool [17]; structure depositor(s), release date and primary publication; structure validation and accuracy assessments; and basic information on the chemical and structural features of the entry. For example, we used these pages to filter through many entries when researching a feature on viral quasi-symmetry (http://pdb101.rcsb.org/motm/200). The “Global Stoichiometry” field [18] underscores one of the mysteries of papillomaviruses that was revealed by the structural biologists (e.g., PDB ID 3j6r [19]). They are “homo-360-mers,” which places a surprising value of 6 subunits in the repeating unit of the icosahedral symmetric virus (Fig. 2). This multiple of 60 (*i.e*., 60 × 6 = 360) does not conform to the classic system of quasi-symmetry, which would require 3, 4, or 7 subunits to be consistent with the conception of a distorted triangular tessellation of a virus this size [20]. The PDB ID 3j6r structure shows that this virus, and similar polyoma viruses such as simian virus 40 [21], instead place pentamers at locations normally occupied by hexamers, and use flexible polypeptide chain segments to resolve the inconsistencies in sites of interaction between pentamers. This information is directly relevant to the design and engineering of second-generation virus-like particles that can be formulated as HPV vaccines to prevent cervical cancers [22].

## Structure and function: HPV form and flexibility

Looking at the dozens of structures available for papillomavirus capsids and individual proteins, we see many of the general themes that underlie biomolecular structure and function. For example, mechanisms of hierarchical assembly and self-association guide the construction of icosahedral capsids, as revealed in structures like that shown in Fig. 2 (PDB ID 3j6r). Transient association of viral proteins with host proteins and nucleic acids guide each step in the viral lifecycle and have been revealed at atomic detail. For example, PDB ID 5w1o [23] includes an L1 pentamer from HPV16 bound to oligosaccharides from the cellular heparin receptor, with the surprising observation that multiple sites of virus-receptor interaction are involved in viral attachment and entry. Intrinsically disordered proteins also play central roles in several intracellular processes, notably the oncogenic interaction of E6 and E7 proteins with disordered segments of host proteins, described in more detail below.

**Fig. 3:**
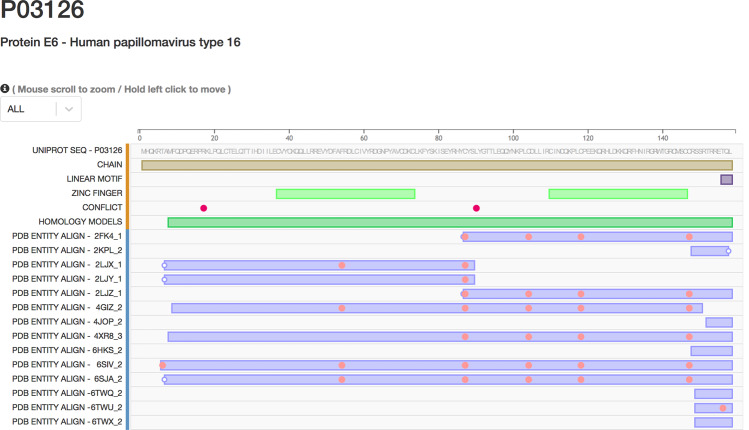
The *Protein Feature* view summarizes all PDB entries for a particular protein. *Protein Feature* view for a complex of HPV16 E6 with guanylate kinase MAGI-1 (PDB ID 2kpl [24]) helps users understand that only a small peptide from E6 is included in the PDB ID. A similar View is available for MAGI-1, showing that only one domain from that protein is included in the entry.

To explore these topics, the RCSB PDB website provides a collection of “Views” that leverage information from related sequence and structural resources, allowing users to drill deeper into the information held in each entry. The *Protein Feature* view, provided in summary form on the *Structure Summary* page and in more detail with one click, gathers data from UniProt and other external databases to assist users in understanding the context of each entry in the PDB archive. Figure 3 shows one major use of the *Protein Feature* view. Structural biologists often cut proteins into functional pieces when the full-length protein does not prove amenable to structure determination in its entirety. It can, therefore, be difficult to parse out exactly which polypeptide chain segments comprising a particular protein are present in a given PDB ID. The *Protein Feature* view for HPV16 E6 shows that the protein contains several functional domains, and structures are available for the whole protein and for two individual domains, as well as for a short peptide bound to the PDZ1 domain of cellular protein MAGI-1 [24].

Similar “views” enable exploration of other important topics. The *Small Molecule* view provides information on ligands bound to macromolecules in each structure. For example, PDB ID 2gxa [25], provides an atomic-level direct look view at the nucleotide binding site of the E1 hexameric helicase assembly bound to segment of single-stranded DNA. The *Annotations* view includes third-party annotations relating to domain structure and gene ontology (e.g., CATH defines the two domains of E1 as zinc fingers, similar in structure to that found in the large T-antigen D1 domain, and a so-called Rossmann fold [26]). Further details of the E1 protein sequence itself may be explored in the *Sequence* view, with annotations of secondary structure and other features, such as the nucleotide binding site. Details concerning the structure determination process are tabulated in the *Experiment* view. Finally, quantitative analyses of structure quality can be found in the wwPDB Validation Report, which is summarized graphically near the top of the *Structure Summary* page and available both in 3D (click 3D Report) and downloadable pdf file (click Full Report).

**Fig. 4:**
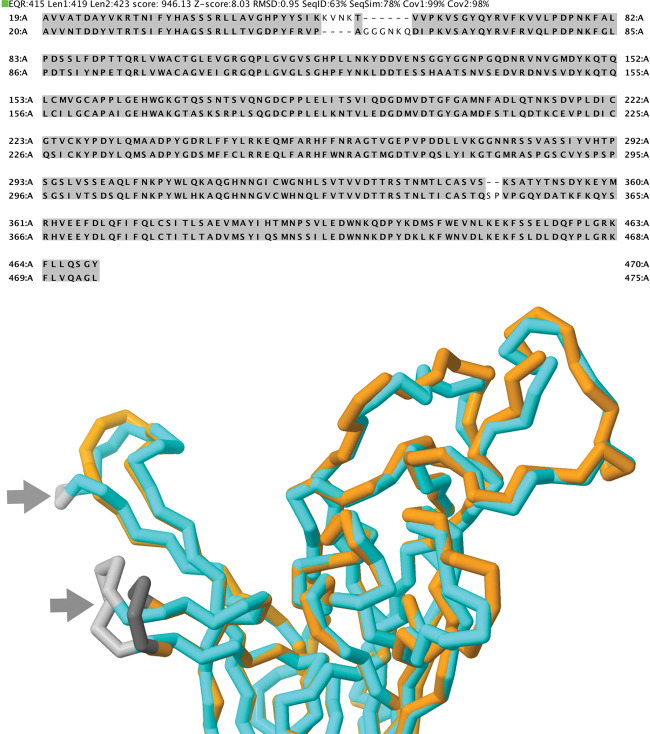
Sequence and structure alignments allow analysis of conservation and flexibility. Comparison of L1 proteins from pentamers of HPV11 (cyan, PDB ID 2r5k [31]) and HPV18 (orange, PDB ID 2r5i [31]), using the Java Web Start “Structure Alignment” tool. Arrows indicate sites with changes in the length of loops, seen as gaps in the sequence alignment at the top.

Structure determinations typically provide “snapshots” of macromolecules adopting a single conformational state. For relatively simple proteins consisting of a single globular domain (e.g., sperm whale myoglobin PDB ID 1mbn [27], the first atomic-level protein structure to be determined [28]), this is not a usually a major consideration. For more complicated macromolecular systems consisting of more than one globular domain (e.g., the multi-domain Abl protein kinase PDB ID 1fpu [29]) or multi-protein complexes (e.g., the CDK2/Cyclin A binary complex PDB ID 1fin [30]) a conceptual model of conformational flexibility must be built up by gathering structures in different states and comparing them. This challenge is further complicated by the fact that structural biologists often gather information from multiple viral strains and multiple host organisms, so the overall framework must be built using 3D structures from disparate sources. The *Protein Comparison Tool*, accessible using Java Web Start (http://www.rcsb.org/pdb/workbench/workbench.do?action=menu), is a critical tool for relating and comparing different structures. It provides several turnkey methods for pairwise sequence and structure alignments. For example, Fig. 4 includes alignment of capsid L1 protein structures from benign and high-risk strains, showing how small changes in the sequences of polypeptide chain loops on the viral surface lead to conformational differences, and ultimately to differences in the way they are recognized by the immune system [31].

## Visualizing functional interactions: oncogenesis and epitopes

Structural biology allows us to overcome the limitations of the human eye to “see” directly the molecular processes that underlie viral oncogenesis and immune response. A wide variety of visualization systems are available to help researchers explore and make sense of such data. These tools include highly optimized stand-alone software packages such as Chimera [32] and VMD [33], which typically have built-in options to fetch structures from the PDB archive based on the PDB ID. The principal limitation of these molecular graphics systems is the need to download software to the user’s computer and keep pace with frequent updates.

**Fig. 5:**
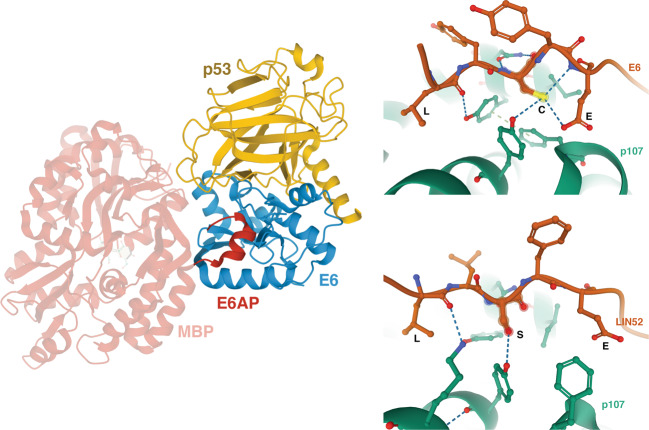
Visualization of virus-host interactions with Mol*. Left: HPV E6 brings together E6AP and p53 tumor suppressor (PDB ID 4xr8 [34]). Only portions of E6AP and p53 are included in the structure determination, and E6AP was studied as a chimera with maltose binding protein. Right: interaction of p107 with E6 and with a suboptimal host partner (PDB IDs 4yos, 4yoz [35]).

The RCSB PDB website provides several options for on-demand visualization, to simplify exploration of structures on-the-fly as the archive is being searched. The most powerful of these tools is the RCSB PDB Mol* web-native molecular visualization tool with many options for customizing views and molecular representations [17]. It has been highly optimized to allow interactive loading and viewing of the large structures that are increasingly being deposited to the PDB archive. In Fig. 5, Mol* was used to explore the mechanisms of oncogenesis in two PDB structures, wherein viral proteins E6 and E7 are frustrating host defenses. HPV E6 acts as an adapter protein, bringing together defense proteins such as the p53 tumor suppressor and E6AP, which leads to recruitment of the ubiquitin/proteasome system. PDB ID 4xr8 [34] illuminates how the LxxLL motif of E6AP is recognized by HPV16 E6, targeting the p53 protein for degradation. Binding of the HPV16 E7 LxCxE motif to the Rb tumor suppressor paralog p107 is seen in a structure of an E7 peptide bound to the protein (PDB ID 4yoz [35]). This interaction blocks the Rb binding site involved in cell cycle signaling, as seen in a complex with LIN52 peptide (PDB ID 4yos [35]). LIN52 has an LxSxExL motif and a phosphorylated serine.

**Fig. 6:**
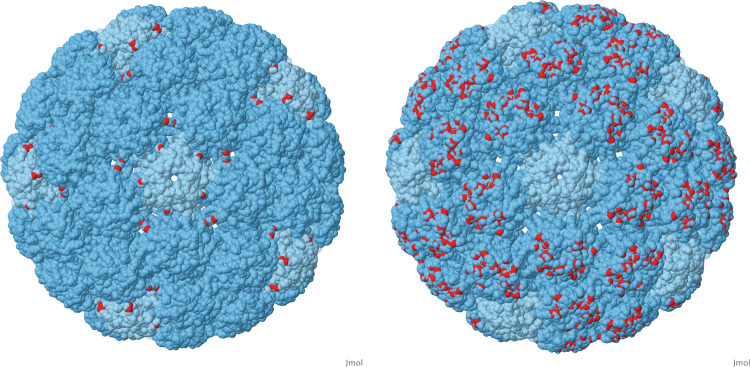
Visualization of epitopes with JSmol. Epitopes (red) of antibodies U4 (left) and V5 (right) on HPV16 capsids, visualized with online JSmol scripting tools (PDB IDs 6bsp, 6bt3 [37]).

JSmol is also provided at the RCSB PDB website as a lighter-weight visualization option with a user-friendly natural language scripting language [36]. This scripting capability was useful for creation of Fig. 6, which shows antibody-binding epitopes for two structures of HPV virus-like particles. Information from the primary reports for two PDB structures (PDB IDs 6bsp and 6bt3) of HPV16 with bound monoclonal antibodies U4 and V5, respectively, was used to define their epitopes [37], and then scripted for display using JSmol. U4 has a discontinuous epitope (red in Fig. 6) occurring in a groove between pentamers at the fivefold axis (lighter blue in Fig. 6) and one of the neighboring quasi-symmetrical pentamers. In contrast, V5 binds at several positions around the quasi-symmetrical pentamers. Antibody-capsid complex structures can be used to guide second-generation vaccine design efforts, building on the success of currently approved anti-HPV vaccines [22].

## Outreach/education: disseminating the results of HPV research

**Fig. 7:**
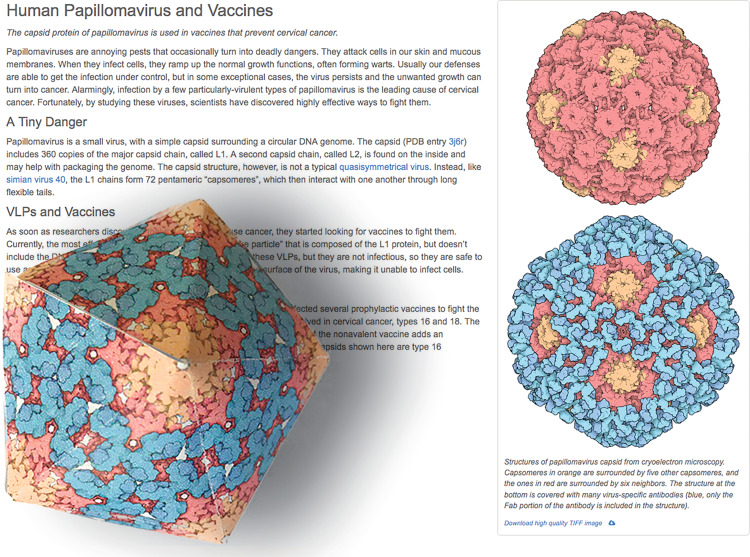
Outreach materials introduce structural topics to a wide audience. Outreach materials include a feature and a foldable paper model of HPV (red) bound to antibody Fab fragments (blue) (PDB ID 6bt3 [37]*).

The PDB archive is a unique resource for science education and outreach, given that structural information provides an intuitive and comprehensible window into difficult functional concepts in biology and medicine. RCSB PDB hosts a web portal, PDB-101 (pdb101.rcsb.org), that brings the results of structural biology to the education and lay communities [38]. As with the diverse user community of the main RCSB PDB portal, the educational and lay communities have a broad collection of needs, so PDB-101 provides multiple modalities for engaging users. A user-friendly browser is provided at the entry point that allows users to explore holdings based on common topics, such as biological energy, nanotechnology, or viruses. The highly popular *Molecule of the Month* feature presents a new topic each month, providing a short description of the structure, function, and relevance of selected molecules [39]. Links to structures in the PDB archive invite users to extend their reading by exploring the actual data. *Curriculum Modules* provide educational materials and lesson plans for popular topics in diabetes, immunology, and virology. In addition, a variety of posters, interactive animations, molecular origami paper-folding activities, and similar materials have been created to engage user communities at all levels of expertise. For HPV, a *Molecule of the Month* feature was recently presented, describing the connection to cancer and how an understanding of HPV proteins can help discover new ways to fight viral infection. A molecular origami foldable paper model of the HPV16 virus-like particle decorated with Fab fragments of the V5 antibody (PDB ID 6bt3 [37]) is downloadable as part of this outreach effort (Fig. 7).

## The RCSB PDB and cancer research writ large

The RCSB PDB is committed to empowering cancer researchers, with advanced tools for exploring biomedically relevant structure holdings, and extensive introductory resources to lower the barrier to entry for users who are new to structural biology (https://www.rcsb.org/pages/help/index). For clinical researchers the RCSB PDB provides easy-to-use tools that enable discovery of cancer-related proteins and biomarkers; support in-depth 3D analyses of mutational hotspots identified via comprehensive genomic sequencing/profiling; and facilitate hypothesis generation regarding selection of targeted antineoplastic agents. The ability to combine 3D structure data with cancer-related information will complement well-established approaches that principally utilize 1D genome/protein sequence data. One of the most important challenges facing clinical researchers today is acquired resistance to targeted antineoplastic agents. Resistance is observed in many tumor types and can appear during any stage of cancer treatment through a variety of biochemical and cell biological mechanisms (reviewed in [40]). The RCSB PDB website also provides tools for understanding acquired drug resistance in 3D and developing testable hypotheses for alternative targeted therapies, as demonstrated for osimertinib treatment failure due to emergence of a previously unreported epidermal growth factor receptor mutation (methionine 766→glutamine) [41]. PDB data and the RCSB PDB website support early-stage oncology drug discovery (reviewed in [6]). Areas of demonstrated impact include target validation; druggability assessment; characterization of screening hits; medicinal chemistry optimization of pharmaceutically acceptable leads; and design of novel proteins for diagnostic and therapeutic applications (e.g., chimeric antigen receptors, bispecific antibodies). Finally, the RCSB PDB website supports basic and applied cancer researchers, whose work can benefit significantly from “direct looks” at 3D structures of human proteins, multi-protein complexes, and protein-nucleic acid complexes as they characterize the biochemical and cell biological origins of human cancers (reviewed in [6]).
